# Epistemology of synthetic biology: a new theoretical framework based on its potential objects and objectives

**DOI:** 10.3389/fbioe.2023.1266298

**Published:** 2023-11-20

**Authors:** Mirco Plante

**Affiliations:** ^1^ Collège Montmorency, Laval, QC, Canada; ^2^ Centre Armand-Frappier Santé Biotechnologie, Institut National de la Recherche Scientifique, Université du Québec, Laval, QC, Canada

**Keywords:** synthetic biology, philosophy of biology, epistemology, natural, non-natural, artificial, virtual, living

## Abstract

Synthetic biology is a new research field which attempts to understand, modify, and create new biological entities by adopting a modular and systemic conception of the living organisms. The development of synthetic biology has generated a pluralism of different approaches, bringing together a set of heterogeneous practices and conceptualizations from various disciplines, which can lead to confusion within the synthetic biology community as well as with other biological disciplines. I present in this manuscript an epistemological analysis of synthetic biology in order to better define this new discipline in terms of objects of study and specific objectives. First, I present and analyze the principal research projects developed at the foundation of synthetic biology, in order to establish an overview of the practices in this new emerging discipline. Then, I analyze an important scientometric study on synthetic biology to complete this overview. Afterwards, considering this analysis, I suggest a three-level classification of the object of study for synthetic biology (which are different kinds of living entities that can be built in the laboratory), based on three successive criteria: structural hierarchy, structural origin, functional origin. Finally, I propose three successively linked objectives in which synthetic biology can contribute (where the achievement of one objective led to the development of the other): interdisciplinarity collaboration (between natural, artificial, and theoretical sciences), knowledge of natural living entities (past, present, future, and alternative), pragmatic definition of the concept of “living” (that can be used by biologists in different contexts). Considering this new theoretical framework, based on its potential objects and objectives, I take the position that synthetic biology has not only the potential to develop its own new approach (which includes methods, objects, and objectives), distinct from other subdisciplines in biology, but also the ability to develop new knowledge on living entities.

## 1 Introduction

Since the discovery of DNA (Watson and Crick, 1953), several techniques and disciplines have been developed to understand, modify, and create new biological entities. Synthetic biology^1^ is a new research field which attempts to accomplish this objective by adopting a modular and systemic conception of the living organisms, based on practices and techniques developed in engineering (electric, mechanics, computer science) and biology (biochemistry, molecular biology, biotechnology) (Hartwell et al., 1999; Knight, 2005). Indeed, on the one hand, synthetic biology would not have been possible without the various discoveries and fundamental technological breakthroughs in biology: PCR (Saiki et al., 1988), genome sequencing (Hunkapiller et al., 1991; Fleischmann et al., 1995), as well as the discovery of genetic regulation systems (Jacob et al., 2005). On the other hand, the foundation of synthetic biology was based, among others, on concepts borrow from engineering, which considers biological entities as modular systems that can be hierarchized, normalized, modified and (de)assembled, like computers with its components (Endy, 2005).

Two achievements marked and characterized the beginning of the era of synthetic biology as a potential new discipline. First, the development of the concept « *biobrick* » (Elowitz and Leibler, 2000; Gardner et al., 2000; Levskaya et al., 2005), based on the physical and mechanistic vision of the concept « living » by Morowitz (1987). Second, the synthesis of an artificial chromosome (genome) introduced into a bacterium, creating the first functional synthetic living organism named *Mycoplasma laboratorium* (Gibson et al., 2008; 2010). These achievements lead to the establishment of one of the main goals in synthetic biology: the creation of the minimal living genome and cell (Glass et al., 2017; Lachance et al., 2019).

The development of synthetic biology has generated a pluralism of different approaches that may lead to confusion with other biological fields, like systems biology (Westerhoff and Palsson, 2004; Kirschner, 2005) and biotechnology (Aguilar et al., 2013), and suggests that synthetic biology might only be an umbrella that brings together a set of heterogenous practices and conceptualizations from various disciplines (O’Malley et al., 2007; Raimbault et al., 2016). That said, some scientists consider synthetic biology as an extension of biotechnology (Koide et al., 2009; Erickson, 2011), while other scientists advocate that synthetic biology is the applied science of biology, like engineering is for physics (Church, 2005; Knight, 2005; Serrano, 2007; Lachance et al., 2019).

I present in this article an epistemological analysis of synthetic biology in order to clarify this new discipline by specifying its particular objects of study and objectives.

First, I present and analyze the principal research projects developed at the foundation of synthetic biology, in order to establish an overview of the practices in this new emerging discipline (section 2). Then, I analyze a scientometric study on synthetic biology to complete this overview (section 3). Afterwards, considering these analyses, I suggest a three-level classification of the object of study of synthetic biology (which are living entities that can be built in the laboratory), based on three successive criteria: structural hierarchy, structural origin and functional origin (section 4). Finally, I propose three successively linked objectives in which synthetic biology can contribute (where the achievement of one objective led to the development of the other): Interdisciplinarity collaboration (between natural, artificial, and theoretical sciences), knowledge of natural living entities (past, present, future, and alternative), pragmatic definition of the concept of “living” (that could be used by biologists in different contexts) (section 5).

In doing so, I take the position that synthetic biology has not only the potential to develop its own new approach (and objects), distinct from other subdisciplines in biology, but also the ability to develop new knowledge on living entities (as objective).

## 2 Plurality of research programs in synthetic biology

**FIGURE 1:**
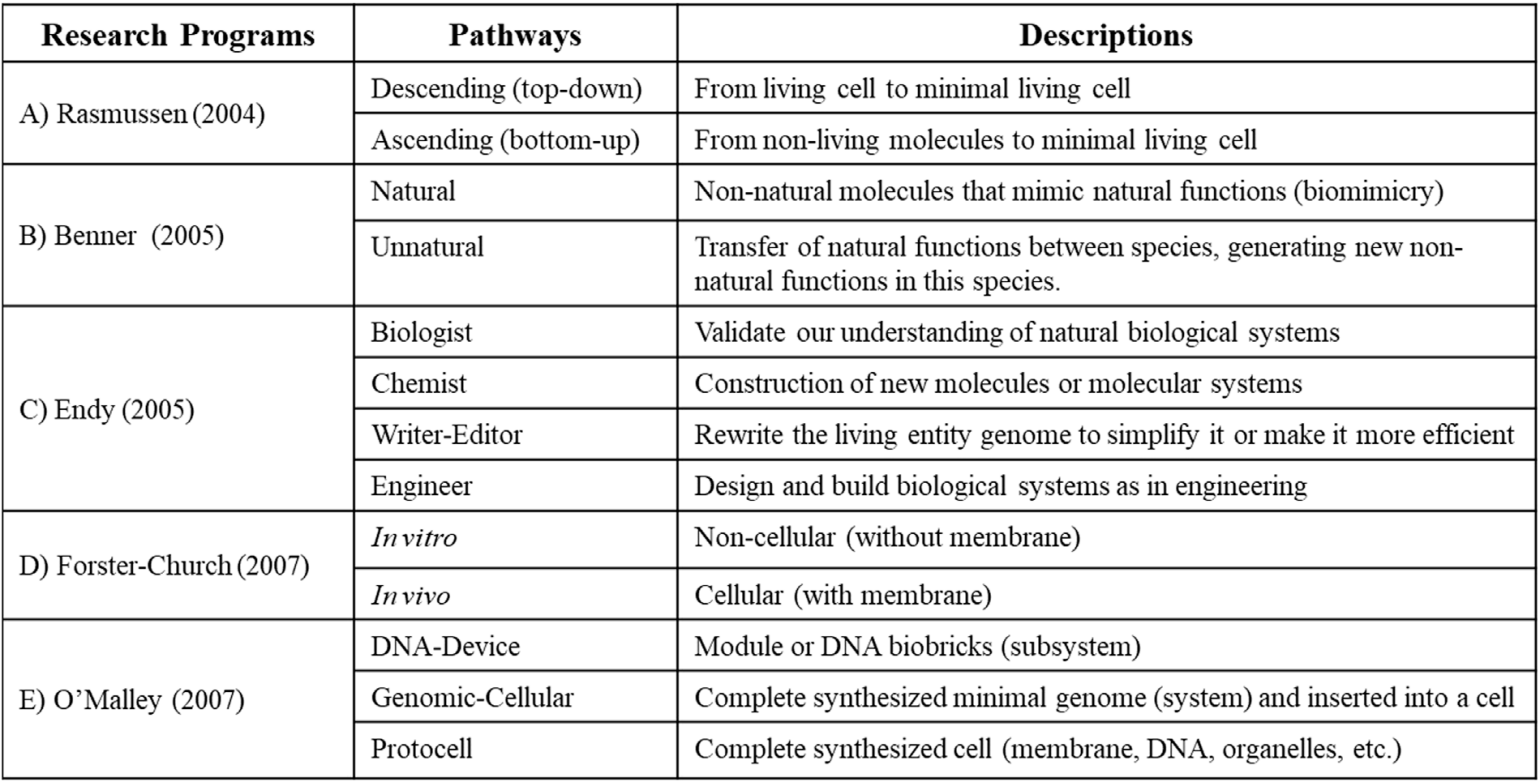
Main “research programs” (composed of different pathways) in synthetic biology. **(A)** Rasmussen *et al.* (2004); **(B)** Benner and Sismour (2005); **(C)** Endy (2005); **(D)** Forster and Church (2007); **(E)** O’Malley *et al.* (2007).

To define the potential discipline of synthetic biology, it is necessary to analyze the different approaches used by its practitioners in order to develop a common “paradigm” or “research program"^2^ (Raimbault et al., 2016, p.10): « *It is crucial to study the way groups of scientists emerge and develop shared visions of their research topic, and eventually create what Kuhn coined as a paradigm* ». I present and analyze in this section five main research projects (or “research programs”) composed of different pathways at the foundation of synthetic biology, which will allow to better define the objects and objectives to this new discipline in the next sections of this manuscript (Figure 1).

### 2.1 Top-down and bottom-up pathways (Rasmussen)

Following the analysis of two important international conferences on synthetic life, the team of Rasmussen *et al.* (2004) proposes a research program composes of two pathways based on methods for building living entities in synthetic biology (Figure 1A). This classification is closely related to one of the main projects of synthetic biology: creating the minimal living cell (Xu et al., 2023).

The “Top-down” (or descendant) pathway attempts to build new living entities by taking as a starting point a simple natural unicellular living organism (with a small genome) that will be reduced to the minimum of its structures and functions following mainly genetic manipulations on their genome, in order to obtain the minimal living cell. For example, the studies on reducing the genome of the bacteria *Mycoplasma mycoides* (Hutchison et al., 1999; Glass et al., 2017), *Mesoplasma florum* (Baby et al., 2013; Matteau et al., 2017) and *Escherichia coli* (Posfai et al., 2006).

The “Bottom-up” (or ascendent) pathway, for its part, attempts to build minimal living cells through the assembly of molecular modules (biobricks) made of protein, DNA, RNA or membrane vesicles. For example, the study on building protocells with a lipid vesicle that could self-replicate (Bachmann et al., 1992; Szostak et al., 2001; Adamala and Szostak, 2013; Matosevic and Paegel, 2013; Drobot et al., 2018) or the building of a combined genetic elements into a functional network (Guet et al., 2002).

Let us emphasize that the “top-down” pathway is more particularly favored over the “bottom-up” pathway by synthetic biologists at the moment, considering the large gaps in our knowledge to understanding how cells work. Indeed, it is easier to build the minimum living cell by stripping a given living cell step by step and piece by piece, rather than trying to build an all-functioning living cell all at once with various molecular pieces (Ostrov et al., 2019). That said, these two methods should collaborate and be complementary in a same research program, considering that in the process of building a living entity we might both adding and removing parts along the way.

### 2.2 Natural and non-natural pathways (Benner)

Benner and Sismour (2005) suggest a research program composed of two pathways, based on the functional and structural origins of the created entities in synthetic biology (Figure 1B).

The “Natural” pathway allows the reproduction of biological functions found in natural terrestrial entities using non-natural structures created in the laboratory (like biomimicry; Breslow, 1972). For example, the studies on new monomers (e.g.,: new nucleotides or amino acids) or polymers (e.g.,: new DNA, RNA and proteins) supporting natural functions (Geyer et al., 2003; Isaacs et al., 2011).

The “Non-natural” (or unnatural) pathway allows the artificial integration of new functions within a natural living entity, by modifying or interchanging natural structures from one entity to another in the laboratory. For example, the studies on genetically modified organisms (e.g.,: bioluminescence in mice; Hadjantonakis et al., 1998) or on the creation of new genomes (Palmiter et al., 1982; Gibson et al., 2009).

Thus, Benner build his Natural and Non-natural functional pathways respectively with non-natural and natural structures. Of course, natural functions are also supported by natural structures (e.g.,: all living things in Nature), and non-natural functions are also supported by non-natural structures (e.g.,: all machines built in engineering). That said, the non-natural function expose by Benner seems to be a natural function (supported by a natural structure, like a known gene) transfer artificially from one natural living entity to another one. We could say that the transferring technic is non-natural, but it could happen in nature in some way following natural evolution process. Could we imagine a non-natural function, not found in nature, that a natural living entity (made of natural and/or non-natural structures) could express following laboratory building? In order to apply these natural and non-natural principles more broadly to all kinds of structures and functions, I suggest a redefinition of the research program of Benner into four pathways, where the “natural” pathways could refer to structures and functions found in Nature, while the “non-natural” pathways could include structures and functions imagined and artificially designed in the laboratory by humans. I will develop and use this classification in section 4 of this manuscript, to identify the objects of synthetic biology.

This revised research program of Benner could be complementary to Rasmussen’s program (Figure 1A), considering that an ascending or descending pathways could either be natural or non-natural in their structures and functions built. It could also be applied to other programs, as I will underline in the presentation of the following research programs.

### 2.3 Biologist, chemist, writer-editor, engineer pathways (Endy)

Endy (2005) suggests a research program for synthetic biology divided in four pathways based on different disciplinary fields (Figure 1C).

First, the “Biologist” pathway uses technological advances and methods of synthetic biology to study and validate our present knowledge of natural biological systems. For example, the studies on rebuilding and studying natural genetic circuits *in vitro* and *in vivo* (Sprinzak and Elowitz, 2005).

Second, the “Chemist” pathway considers synthetic biology as an extension of synthetic chemistry, which allows the construction of new molecules for the purpose of drug development or to better understand living entities. For example, the studies on the synthesis of new molecules that can reproduce natural functions, or studies on transferring functions from a given entity to another (Geyer et al., 2003; Isaacs et al., 2011).

Third, the “Writer-editor” pathway rewrite genomes, like a linguist or a computer programmer, to generate living entities that are simplified or more efficient than natural entities for certain functions. For example, we could include in this path the study of redesigning the bacteriophage T7’s genome (Chan et al., 2005).

Fourth, the “Engineer” pathway design and build biological systems in a simpler and more efficient way. For example, we could include in this path the concept of biobricks or modules which allows the building standardization of new entities (Shetty et al., 2008).

Let’s point out a few remarks on the research program of Endy. First, we can see that some pathways within this research program overlap. Among others, the “chemist”, “writer-editor” and “engineer” pathways all have the objective of creating new molecules or biological subsystems. Furthermore, I do not believe that the “biologist” pathway is the only pathway that can develop knowledge about natural biological systems. Indeed, I take the position that all four Ender’s paths can lead to the development of this knowledge in a complementary manner, through their potential to construct module and whole living entities (natural or non-natural). I will develop this point of building and knowing in section 5 of this manuscript, where I expose the fundamental objectives of synthetic biology.

Then, we note that Endy’s program develops different pathways in connection with other disciplines (biology, chemistry, programmer-computer scientist, engineering), thus questioning the distinction of synthetic biology from other disciplines, and reinforcing the “multidisciplinary umbrella” label attached to synthetic biology.

Finally, we find similarities between Endy’s and Benner’s program (Figure 1A), where all pathways of Endy could be represented in the “natural” and “non-natural” pathways developed by Benner.

### 2.4 *In vitro* and *In vivo* pathways (Forster-Church)

Forster-Church’s team (Forster and Church, 2007) develop a research program composed of two pathways based on structural hierarchical level of complexity (Figure 1D).

The “*in vitro*” pathway builds autonomous biochemical systems without a cellular membrane. For example, the studying on creating *in vitro* molecular systems composed of DNA (Tian et al., 2004), RNA (Wang, 1984) and/or proteins (Zhang et al., 2004).

The “*in vivo*” pathway build new biological entities at the cellular hierarchical level (bacteria mostly) by reducing, modifying or redesigning their genome (Hutchison et al., 1999; Posfai et al., 2006; Baby et al., 2013; Glass et al., 2017; Matteau et al., 2017).

Let highlight a few points of discussion on the Forster-Church’s program. First, the “*in vitro*” and “*in vivo*” pathways of Forster-Church’s program are similar respectively to the “bottom-up” and “top-down” pathways of Rasmussen’s program, considering that they involve respectively molecular and cellular structural hierarchical levels of life. They are also similar to the different disciplinary paths of Endy’s program, since they involve both “*in vitro*” (pathways chemist, editor-writer, engineer) and “*in vivo*” (pathway biologist) aspects.

Also, the “*in vitro*” and “*in vivo*” pathways seems very similar to each other considering that they operate concretely on the same hierarchical level of complexity: DNA molecule. Indeed, the “*in vivo*” approach focuses on modifying genomes and testing them in a cell, while the “*in vitro*” approach concentrate on DNA segments linked to RNA and protein expression.

Finally, Forster-Church’s program pathways could be complementary with the revised program of Benner, considering that the “*in vitro*” and “*in vivo*” can generate entities from both natural and non-natural structures and functions.

### 2.5 DNA, genome, protocellule pathways (O’Malley)

O'Malley and Dupré (2007) proposes a research program composed of three pathways based on both the structural hierarchy of the entities and the techniques to be used in the building process (Figure 1E).

First, the “DNA-device construction” pathway aims at building modular biological components (biobricks, modules or genes) using a “bottom-up” method (Wang, 1984; Tian et al., 2004; Zhang et al., 2004; Chen and Elowitz, 2021).

Second, the “genomic cell engineering” pathway aims to synthesize minimal genomes and insert them into genome-less cells. To do this, genomes would first be generated theoretically by bioinformatics and then constructed in the laboratory following “bottom-up” (Palmiter et al., 1982; Guet et al., 2002; Gibson et al., 2009) and “top-down” methods (Hutchison et al., 1999; Posfai et al., 2006; Baby et al., 2013; Glass et al., 2017; Matteau et al., 2017).

Third, the “creation of a protocell” pathway has the objective of creating a complete functional cell from scratch. To do this, the cell would be constructed by assembling cellular subsystems (modules) and membrane vesicles using the “bottom-up” method (Bachmann et al., 1992; Szostak et al., 2001; Adamala and Szostak, 2013; Matosevic and Paegel, 2013; Drobot et al., 2018).

Let’s raise a few points of similarities between the program of O'Malley and the other programs. First, at the level of experimental techniques and methods, “Device-DNA construction” and “creation of a Protocell” pathway correspond to the “bottom-up” pathway of Rasmussen’s program, while “genomic cell engineering” pathway corresponds to the “top-down” pathway of Rasmussen’s program.

Second, at the level of molecular hierarchical level of complexity, the “gene” pathway corresponds to the pathways “*in vitro*” of Forster-Church and “bottom-up” of Rasmussen. On the hierarchical cellular level of complexity, the “genome and cell” pathway corresponds to the pathways “*in vivo*” of Forster-Church and “top-down” of Rasmussen. That said O'Malley develops a more detailed, flexible and complementary gradation of these hierarchical pathways than those presented by Forster-Church and Rasmussen.

Finally, we also note that O'Mailey’s program is complementary to Benner’s program (as are those of Forster-Church and Rasmussen), considering that its different paths of construction generate entities that can be “natural” or “non-natural” for their structures and functions.

Considering this analysis of the different research programs in synthetic biology, we could develop a more general and common research program based, on the one hand, on the structural hierarchical level of the constructed entity (or object of study), similar to the programs of O'Malley, but with a broader scope to include entities beyond the cellular level. On the other hand, we could develop a research program based on the structural and functional origin of the constructed entity (or object of study), like the revised program of Benner exposed previously.

In doing so, we should not define a research program based on laboratory techniques (e.g.,: Forsters-Church’s “*in vivo*” and “*in vitro*” pathways, Rasmussen’s “bottom-up” and “top-down” pathways, or Endy’s disciplinary pathways) considering that they could be modified over time and eventually become obsolete due to technological advances. That said, all research projects (and pathways) should use and share different methods and techniques in a complementary and collaborative process to achieve its objectives, as proposed by the research program thesis of Lakatos, 1968-1969 (see footnote 2). I will present in the last two sections a new research program for synthetic biology, by defining its objects and objectives, that could address the problems mentioned in this section and guide the development of this discipline to its full potential.

Before presenting my research program proposal, I analyze in the following section a scientometric study of synthetic biology based on O'Malley’s program, which allows an in-depth analysis of all the research published since its foundation, in order to complete the overview of this new discipline.

## 3 Scientometric study on synthetic biology

Following the advancement of research, techniques, and scientific publications in the field of synthetic biology, scientometrics uses different computerized tools to process and present the results of analyses made from banks of scientific publications. This thorough process could help to establish a research program (or paradigm) for a given discipline.

An important scientometric study, published in 2016 by Raimbault et al. (2016), analyzes 4,605 articles on synthetic biology published between 1980 and 2015 (from the Thomson Reuters Web of Science article bank)^3^. I present in this section two characteristics of synthetic biology revealed by this scientometric study: modulation and heterogeneity.

### 3.1 Building new modules and systems

**FIGURE 2:**
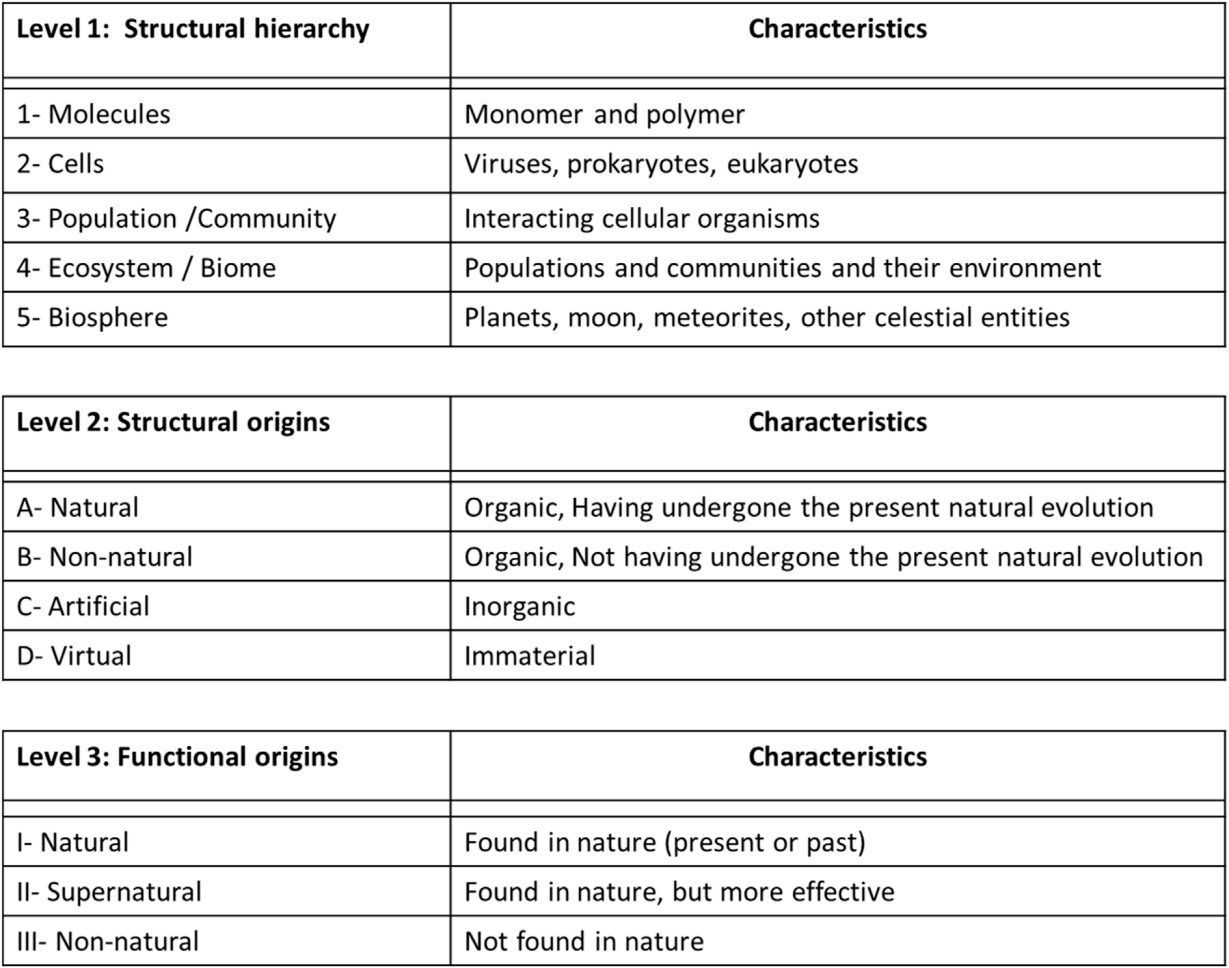
Classification of the objects created by synthetic biology based through three successive criteria of construction: Structural hierarchy (1–5), Structural Origins **(A-D)**, and Functional Origins (I and III).

The study analyzes synthetic biology according to a classification divided into 4 methodological categories. The first three categories are directly linked to O'Malley’s research program (Figure 2E): “biobrick engineering” (corresponding to “DNA-device construction” of O’Malley), “genomic engineering” (corresponding to “genomic cell engineering” of O’Malley) and “protocell creation” (corresponding to “protocell creation” of O’Malley). The fourth category adds to O'Malley’s classification, named “metabolic engineering”, pursues the idea of building small cell-based industries with the construction of new metabolic pathways to produce a desired molecule, such as drugs (Ro et al., 2006) or biofuels (Peralta-Yahya et al., 2012; Choi and Lee, 2013). As the authors themselves point out in their studies, this category is in fact only the application of the three other categories of Raimbault’s classification (or O’Malley’s), leading us to doubt the relevance of this addition. Moreover, this path seems to only takes up the torch of biotechnology, following commercial and practical type of research to produce molecules for human purpose and needs.

That said, of these four categories, the authors assert that the pathway of “biobrick engineering” would be the most important at the foundation of synthetic biology, considering its specific potential to create new modules (biobricks) or whole systems (living entities) not found in nature. In doing so, this pathway casts a new and innovative light on the study of natural living entities and on scientific practices in the laboratory not found in other disciplines. This new avenue could contribute to the development of new knowledge on living entities, as I will expose in the last section of this manuscript.

### 3.2 Heterogeneity

The scientometric study also points out a general heterogeneity within synthetic biology, suggesting that this discipline is not a new science *per se*, but rather an umbrella that encompasses an amalgam of techniques and concepts. In doing so, the authors assert that synthetic biology has for the moment only potential for technological innovation, through its capacity to build biological entities. That said, this heterogeneity might heralds the emergence and stability of this new discipline in the making.

I would add to their affirmation that the plurality and diversity of propositions should be encouraged when brainstorming ideas. But over time, they can become confused if they are not analyzed, organized and articulated with one another, as part of a main comprehensive plan of actions. Thus, I’m for an organized heterogenous discipline, where the diversity of the propositions (e.g.,: methods, objects, objectives) is analyzed on their similarities, differences, and complementarities, in order to articulated them as a whole (e.g.,: a main research program). The present manuscript is a respond to establish a more articulated and heterogenous research program for synthetic biology, based on its object and objectives.

Following this assertion, let us analyze the heterogeneity of synthetic biology which, as I will demonstrate, seems for the moment to be more homogeneous than the authors of the article suggest.

First, the study reveals that the core of synthetic biology was developed mainly in the United States (publications, institutions, scientists)^4^ and plays the role of boundary spanners at the foundations of this discipline, revealing a more homogeneous than heterogeneous origin of synthetic biology. Although we can undoubtedly consider the United States to be the cradle of synthetic biology, it will be necessary to follow and consider the development of synthetic biology elsewhere in the world before we can depict a more heterogeneous picture of the discipline, as the (European commission, 2005, p.5) pointed out: « *Synthetic biology is a nascent field, and there is currently no systematic, global effort to coordinate the developments in this field. Much of the research so far has been pioneered by individual groups in the US, and the European research community has been relatively slow to embrace the field. What is needed [.] is a framework for coordinating the current research, fostering a community of researchers [.] and creating a forum for the establishment of clear goals, shared tools and agreed standards* ».

Secondly, the authors use three “Indicators” to select the articles in the scientific literature in order to describe and define synthetic biology: core institutions, business development, governance. The “business” (or commercial) indicator, which selects, for example, research on the production of drugs, biofuels and cosmetics through synthetic biology, is particularly strong in this selection process, as the authors themselves underline (Raimbault et al., 2016, p.14-15): « *The indicator “business development” was built from three kinds of information: participation in a Scientific Advisory Board (SAB), creation of a start-up, and ownership of patents as inventors […] The “business development” indicator is particularly strong compared to the other two indicators. The proximity of members of the core-set with commercial activities is almost systematic, suggesting that the relationships between academia and industry are particularly structuring* ». In doing so, this criterion orient and homogenizes their scientometric study towards an economic aspect, reinforcing again the idea that synthetic biology is merely an extension of biotechnology. For my part, as I will expose in the last section of this manuscript, I prioritize an objective that is more fundamental than economics, in order to establish a broader, objective, heterogeneous and collaborative discipline that has the potential to develop new knowledge on living entities (rather than economic aspects).

Finally, the scientometric study concludes that the specific object of study in synthetic biology is mainly located at the DNA level, associated with the used of the terms: genomes, biobricks, genetic networks and circuits^5^ (as also observe with the different research programs exposed in the previous section of this manuscript; Figure 2).

That said, focusing only on the DNA molecules (or proteins) might seem reductive and homogeneous for the object of study of synthetic biology, considering the different structural levels of the hierarchy in Nature (e.g., virus, unicellular and multicellular entities, populations, communities, ecosystems, biomes, biospheres), as well as the diversity of the biological entities on each of this level. In order to widen and heterogenized the field of study of synthetic biology, we should consider extending its object of study to other hierarchical levels of complexity of living organisms. In doing so, it could help to better define this new discipline. Indeed, a discipline can be defined in different ways depending on the point of view chosen: academic institutions (Becher, 1989; Turner B. S., 2000; Parker, 2002; Aram, 2004), social structures (Apostel et al., 1972; Huber and Shaw, 1992), the object of study (Boisot, 1972; Squires, 1992). This scientometric study exposed that synthetic biology has developed a diversity of research programs that provide the singular ability to construct its own object of study (living entities) following different methods. Considering this particularity, I support the idea of defining a discipline by its object of study rather than its academic and social structures.

For example, Squires (1992, p.202) suggests three “dimensions” to define a discipline through its object of study: *« i) what they are about (object, content, topics or problems); ii) their stance toward that object, in terms of a concern with knowing, doing or being (methodologies, techniques and procedures); and iii) the extent to which they are operating in a normal, reflexive or philosophical mode (in the extent to which the discipline treats its own nature as the subject of reflexive analysis)* »*.* In the case of synthetic biology, the “object dimension” could be the living entities (as I will expose in section 4 and Figure 2), the “method dimension” could be linked to its ability to build all kinds of living entities in laboratories following different laboratory technic of construction (as expose in section 2 and Figure 1), and the “philosophical dimension” could be linked to the analysis of the orientations and objectives of synthetic biology (as I will expose in section 5).

Thus, following this scientometric study and the research programs expose in the last section (Figure 2), we observe that synthetic biology is for the moment homogenous in its origins, objects of study and objectives. These characteristics might reflect the “child” stage of this discipline prior to its implementation (Bensaude Vincent, 2013, p.128).

That said, the development of its locations (countries) and technologies (methods), as well as its specific objects and objectives, could reveal a more heterogeneous discipline, which could then be analyzed (on its similarities, differences and complementarities) in order to articulate a comprehensive core research program for synthetic biology.

I present in the next two sections of this manuscript some thoughts on the objects and objectives of synthetic biology that might help this potential new discipline to pass from “child” to “coming of age” state of development, as an articulated heterogenous discipline.

## 4 Structural and functional objects of synthetic biology

I present in this section a proposition of classification of the objects of study built by synthetic biology based on three successive criteria, inspired mainly by the “research program” of O’Malley (Figure 1E; which shares similarities with the research program of Rasmussen, Endy and Forster-Church) and Benner (Figure 1B; which is complementary to all other research programs): Structural hierarchy, Structural Origins, and Functional Origins (Figure 2). This classification of objects could help define synthetic biology as a discipline, and precise its objectives (as I will expose in section 5).

### 4.1 Structural hierarchy

First, inspired by the research program of O'Malley (Figure 1E), we find a criterion that classifies the object of study according to its structural hierarchy of complexity found in Nature (Figure 2, Level 1, hierarchies 1–5): molecule (e.g.,: monomers and polymers of protein, carbohydrate, lipid and nucleic acid), cell (e.g.,: virus, prokaryote, eukaryote), population-community (e.g.,: grouping of several unicellular and/or pluricellular entities), ecosystem-biome (e.g.,: population and/or community of cellular organisms and their environment) and biosphere (e.g.,: a whole planet, meteorite, stars or other celestial objects)^6^.

As criticized previously following the analysis of the research programs and scientometric analysis, synthetic biology limits for the moment its object of study mainly to the level of molecule (Figure 2, Level 1, hierarchy 1; e.g.,: DNA fragments, genomes, gene circuits) and unicellular organism (Figure 2, Level 1, hierarchy 2; e.g.,: lipids cellular membranes). Thus, this first criterion broadens the scope of this new discipline to include different structural levels beyond the unicellular organism, as exposed with the following levels of structural hierarchy.

At population and community level (Figure 2, Level 1, hierarchy 3), we could study the communications and interactions between the cells of a given cellular population or communities (Yokobayashi et al., 2003; Tamsir et al., 2011; Cable et al., 2022), involved in the repair, modification, replacement, or addition of cells to the whole. For example, at the bacterial level, scientists are trying to recreate communication systems (“quorum sensing”) to develop new bacterial populations and communities (Brenner et al., 2007; Jaramillo, 2017). We could also study the possibility for a unicellular population to form a pluricellular organism by identifying the genes that allow this transition in Nature, such as the study on the amoeba *Dictyostelium discoideum* that identified 4 genes essential for this process (Wang et al., 2021).

At the ecosystem level (Figure 2, Level 1, hierarchy 4), we could repair, modify, or replace our terrestrial ecosystems and biomes to ensure their balance and prosperity on our planet. Indeed, human activity is currently altering and deteriorating natural terrestrial ecosystems, causing, among other things, a sixth mass extinction of living species (Ceballos et al., 2017). This extinction will eventually jeopardize certain keystone species supporting ecosystems, potentially leading to their collapse (Paine, 1995; Davic et al., 2003). Scientists could thus intervene on these keystone species to preserve the ecosystem, such as the study of the kangaroo rat *Dipodomys* replaced by the mouse *Chaetodipus baile* in the Chihuahuan Desert in the United States (Brown and Heske, 1990; Ernest and Brown, 2001).

At the level of an entire biosphere (Figure 2, Level 1, hierarchy 5), considering again the imbalances caused by human activity, scientists could intervene to repair and modify our biosphere to preserve its balance and prosperity (Solé et al., 2015; Griscom et al., 2017; Solé and Levin, 2022). For example, we could intervene in the terrestrial geobiochemical cycles, such as the CO2 cycle responsible for global warming (Delisi, 2019; Delisi et al., 2020), or in the web of biodiversity (Redford et al., 2013). We could also imagine 1 day being able to reproduce the terrestrial biospheres (by terraforming process) on other planets, moons or celestial objects, following the successive integration and coordination of biological functions supported by different living species (Menezes et al., 2015; Conde-Pueyo et al., 2020).

### 4.2 Structural origins

Then, based on the revised Benner’s research program (Natural and Non-natural pathways, Figure 1B) we find a criterion that classifies the object of study according to its structural origin. This classification is also inspired by the field of Artificial Life, which has similar and complementary objectives and practical issues to synthetic biology, such as the building of living entities (from artificial and virtual structures) and the extraction of the concept of “minimal life” (Langton, 1989, p.1): « *Artificial life is the study of artificial systems that exhibit behavior characteristic of natural living systems. It is the quest to explain life in any of its possible manifestations, without restriction to the particular examples that have evolved on earth. The ultimate goal is to extract the logical form of living systems* »*.* The philosopher Bedau (2007) goes in the same direction by proposing a classification of the artificial living entities in three categories: soft (e.g.,: computer simulations or digital constructions), hard (e.g.,: mechanical, electronic and computer parts), wet (e.g.,: biochemical systems made in laboratories). Considering the classifications of Benner and Bedau, I suggest four kinds of structural origin for the entities made in the laboratory (Figure 2, Level 2, structures A to D): natural, non-natural, artificial, virtual. In doing so, every structural hierarchy level exposed in the first criteria (Figure 2, Level 1) could be made from any of these four kinds of structure.

First, the entity could be built with “natural” organic structures (Figure 2, Level 2, structure A), made in Nature through evolution process. For example, the study of reconstruction of the entire genome of an entity, both those present in Nature today (Palmiter et al., 1982; Gibson et al., 2009; Gibson et al., 2010) and those past or extinct (e.g., the mammoth; Noguchi et al., 2012). We could also add to this category alternatives entities (that could have been built in nature following possible different evolution paths), future entities (anticipated or predicted evolutionary entities) and minimal entities (which is at the center of all natural living entities, as I will develop in the last section of this manuscript), build in laboratory through modification of natural organisms. We might also be able 1 day to build a hybrid living entity made of mixed parts from earth and exoplanetary, which naturally evolve on their respective planets (e.g.,: by using organic structures found in meteorites; Furukawa et al., 2019). At another structural hierarchical level, we could include the rehabilitation or integration of a natural species from a given community or ecosystem into the biodiversity web of another community or ecosystem (Mooney and Hobbs, 2000; Ernest and Brown, 2001). Let us also highlight this interesting study that attempts to encapsulate in a single cell the metagenome and metabolic network of an entire ecosystem (Belda et al., 2021), which could represent a way to study some aspects of the ecosystems in the laboratory, and ultimately distilled the minimal functional metagenome possible that support an ecosystem.

Second, the constructed entity could be built with “non-natural” organic structures (Figure 2, Level 2, structure B), which are not made in Nature from the evolution process. For example, the studies on the synthesis of new organic molecules (nucleotides, amino acids or hybrid molecules; Geyer et al., 2003; Isaacs et al., 2011), new replicative lipid vesicles (Zepik et al., 2001; Noireaux and Libchaber, 2004), new genes (Palluk et al., 2018) or even new cellular entities from scratch.

Third, the constructed entity may be built with “artificial” inorganic structures (Figure 2, Level 2, structure C). These man-made structures (made of metallic and/or plastic parts) are mechanical, electrical and computer circuits that mimic natural functions found in living entities. For example, nanorobots (small robots at the nanoscale) could act as cellular organelles, such as mitochondria to produce cellular energy, or as a kind of cell in a pluricellular organism, such as lymphocytes to kill cancer cells or pathogens (Esteban-Fernandez de Ávila et al., 2018; Wavhale et al., 2021). At another level of hierarchy, artificial entities could mimic natural pluricellular animals, such as pollinating insects, to alleviate the environmental problems of animal and plant biodiversity loss (Chen et al., 2019). And let’s not forget the thousand and one humanoid robot projects (robot sapiens) developed all over the world, such as the Atlas robot by the company Boston Dynamics^7^.

Finally, the constructed entity may be made of “virtual” immaterial structures (Figure 2, Level 2, structure D). This category could include *in silico* logical models or software systems that can support properties of the living, as Benner and Sismour (2005, p.542) underline: *« Various types of artificial life that live in silico have been suggested as being a form of ‘synthetic biology’. This approach involves using simulations to evolve computational analogues of the emergent behaviours of living systems. Many of these artificial life forms compete for resources (such as computer processor cycles) within a computer, and therefore evolve »*. For example, computer programs that would enable the design of virtual cells (Tomita et al., 2009; Schellenberger et al., 2011; O'Brien et al., 2015; Breuer et al., 2019) or artificial intelligence supporting living properties (e.g.,: AlphaGo developed by Silver et al., 2018).

### 4.3 Functional origins

Finally, once again inspired by the revised program of Benner (Natural and non-natural; Figure 2B), we find the criterion that classifies the object of study according to its functional origin (Figure 2, level 3, functions I to III): natural, supernatural, non-natural. In doing so, every hierarchical structure exposed in the first criteria (Figure 2, Level 1), made from all kinds of structural origins as mentioned in the second criteria (Figure 2. Level 2), could support any of these functions.

First, the constructed entity could perform a “natural” function (Figure 2, Level 3, function I), which has been developed in Nature following the evolution of biological entities. This function may be found originally in a natural entity that developed it following natural evolution (or be transferred to another species following laboratory techniques) or supported by non-natural or artificial entities. For example, the bioluminescence produced by the green fluorescent protein, which is originally expressed by fireflies and jellyfish, can be transferred to other animal species, such as plants (Hu and Cheng, 1995) or animals (Hadjantonakis et al., 1998). At another structural level, we could include studies on the integration of a new species in a given ecosystem, which replace the natural function of an extinct species (Mooney and Hobbs, 2000; Ernest and Brown, 2001). We could also include robots that replace natural insects for the natural function of polinization (Chen et al., 2019).

Second, the constructed entity could perform a “supernatural” function (Figure 2, Level 3, function II), which correspond to a perfected or extrapolated natural function. This category echoes the statement of Morange (2009, p.23) that synthetic biology could perfect or optimize Nature: « *Their conviction is that ‘nature is imperfect and should and can be revised and improved’».* For example, we could include the studies of building entities that produces a more efficient and strong bioluminescence than that found in nature (Cubitt et al., 1995; Heim and Tsien, 1996), or develop a hemoglobin protein that transports oxygen more efficiently in red blood cells (Cooper et al., 2019; Morita et al., 2021), or extend the natural lifespan of a given living cell (Smith et al., 2018; Lan et al., 2019; Mahmoudi et al., 2019). We could also include studies on the design of artificial intelligence (or A-life) that can perform better human intellectual function (e.g.,: *AlphaZero* play the games of Chess and Go better than humans; Silver et al., 2018; McGrath et al., 2022) or human physical capacity (e.g.,: the promising *Atlas* robot from Boston Dynamics; *DIANA* robot that can ski a big slalom, Park et al., 2022; also the team of robots playing soccer; Yu et al., 2023) or footbal (Song et al., 2022).

Third, the constructed entity may perform a “non-natural” function (Figure 2, Level 3, function III), which is not found in nature, but was generated by the human mind and concretized in the laboratory, as underlined by the *New and Emerging Science and Technologies* committee of the European Commission (2005), (p.5): “*For some, synthetic biology is the engineering of biology: the synthesis of complex, biologically based (or inspired) systems, which display functions that do not exist in nature* ". This type of function allows for the exploration and extension of the functional limits of living entities, as O'Malley underline (2007, p.63): « *Instead of being an end, the production of unnatural functions by engineering can be framed as a profound question about biological plasticity and how our understanding of natural phenomena can be extended* ». For example, the production of a new drug by cells created in the laboratory (Bashor et al., 2022), or a bacteria expressing a new metabolic pathway conceptualized in the form of a module capable of degrading a polluting agent in the environment, like plastic or a particular toxic compound. We could also include the creation of a potential entity that can generate its energy from galactic rays (as suggest the bacteria *Desulforudis audaxviator*, which is powered by radioactive substances; Chivian et al., 2008; Atri, 2020). Pushing the imagination a little further, we could build a living entity that can spit fire, in the manner of the myth of dragons.

According to this categorization, a living entity constructed in laboratories thus comprises a hierarchical structural level (molecule, cell, population-community, ecosystem-biome, biosphere), a structural origin (natural, non-natural, artificial, virtual) and a functional origin (natural, supernatural, non-natural). For example, synthetic biology could build a cellular entity (Structural hierarchy level 2: cell; e.g.,: a bacterium), with a non-natural structure (Structural origin level B: non-natural; e.g.,: biobricks or modules made from modified nucleotides and phospholipids that do not exist in nature), which express a supernatural function (Functional level 2: supernatural; e.g.,: powerful bioluminescence).

Moreover, a combination of these classify structures and functions are also possible. Indeed, we could build a structural hybrid entity with different hierarchical levels (molecules and/or cells and-or population-communauté and/or ecosystem and/or biosphere), using different structural origin (natural and/or non-natural and/or artificial and/or virtual). For example, we could build a natural multicellular entity (an animal) to which an artificial molecular module or cell or organ has been grafted (e.g.,: the pacemaker in a heart, a mechanic heart, nanorobots in blood with immune cells functions, or the Neurolink project on human cortex (Musk, 2019)). We could also imagine a biofilm of bacteria (Watnick and Kolter, 2000) in symbiosis with nanorobots, or an artificial organism that could replace a keystone species in a natural ecosystem (e.g.,: a pollinating robot insect (Chen et al., 2019)).

The constructed entity may also have a hybrid function, such as integrating a multicellular entity with a non-natural function into a natural ecosystem performing natural functions. For example, a bacterium with a new module which produces a new molecule that could neutralize a toxic molecule in the natural geochemical cycle of an ecosystem.

Thus, this classification of the objects of construction and study of synthetic biology demonstrates the potential of this discipline to go beyond the construction of DNA or cellular entities as an object of study, with a variety of structural and functional origins. These objects of study could help define the objectives of this new discipline in development, as I will expose in the next section.

## 5 Fundamental objectives of synthetic biology

Following the analysis of the research programs and the scientometric study in synthetic biology, we note that the fundamental objectives of this potential discipline are not clearly established for the moment. That said, based on the classification of the objects of synthetic biology I suggested in the previous section, I propose in this section three successive fundamental objectives for synthetic biology, where one objective brings the other following this sequence: Interdisciplinary collaboration, Knowledge on living entities, Definition of the concept of “living".

### 5.1 Interdisciplinarity collaboration

Each discipline has its specific object of study, objectives, and methods, but in order to develop knowledge on complex objects or questions, we sometimes need to go beyond the classic border of disciplines, toward an interdisciplinarity collaboration (Rossini and Porter, 1979; Choi and Pak, 2006; Jacobs and Frickel, 2009; Brigandt, 2013; Clark and Wallace, 2015; Hannon et al., 2018). That being said, we should not in the process reduce complex problems to disciplines studying lower levels of phenomena by supposing that they are explanatorily more valid or fundamental, like chemistry and physics levels (Oppenheim and Putnam, 1958; Brigandt, 2010, p.309)^8^. In our present case, the “living entity” is a complex object which should not be reduced to its molecular aspects with the DNA molecules (or genes and genomes) studied by molecular biology, as exposed with the analysis of the research programs in synthetic biology (Figure 1). This research process should rather involve a collaborative network of different teams and projects with a specific common object of study (living entities) and a common objective of developing new knowledge on it (this point of view is, among others, based on the theory of knowledge “research programs” developed by Lakatos (1968-1969); see footnote 2 in this manuscript). In doing so, all structural levels of complexity of the living, through an interdisciplinary collaboration between different sub-disciplines of biology (e.g.,: molecular biology, cellular biology, ecology, and synthetic biology), should be involved in developing knowledge on this complex object (as I exposed with my classification of the objects built in synthetic biology; Figure 2, Level 1).

Moreover, as presented previously, synthetic biology contains aspects of several disciplines at its foundation: biochemistry, molecular biology, chemistry, biotechnology, genetics, engineering, computer science, microbiology, biophysics, mathematical and computer biology (Raimbault et al., 2016; Shapira et al., 2017). Thus, synthetic biology is interdisciplinary both internally, considering its foundations, and externally, considering its objects of study shared with other disciplines. In doing so, synthetic biology must, as a first fundamental objective, develop and maintain an interdisciplinary collaboration. As I will demonstrate in this section, this collaboration should go beyond biological disciplines, to include artificial and theoretical sciences.

In order to articulate the interdisciplinary collaboration to study living entities, we could follow the example one of the most cited organizational systems for academic disciplines: the “Three dimensions” classification of Biglan (Biglan, 1973a; Biglan, 1973b; Stoecker, 1993; Simpson, 2017). In the first dimension, Biglan divides disciplines into “hard” disciplines (i.e.,: natural sciences), which “*subscribed to by all members of the field*” (Biglan, 1973a, p. 201), and “soft” disciplines (i.e.,: humanities/social sciences), which “*content and method tend to be idiosyncratic*” (Biglan, 1973a, p. 202). In the second dimension, Biglan classifies more specifically the disciplines into “pure” (e.g.,: mathematics, chemistry, geology … ) and “applied” (e.g.,: engineering). In the third dimension, Biglan separates disciplines engage with “living systems” (e.g., biology) and “non-living systems” (e.g.,: history)^9^.

**FIGURE 3:**
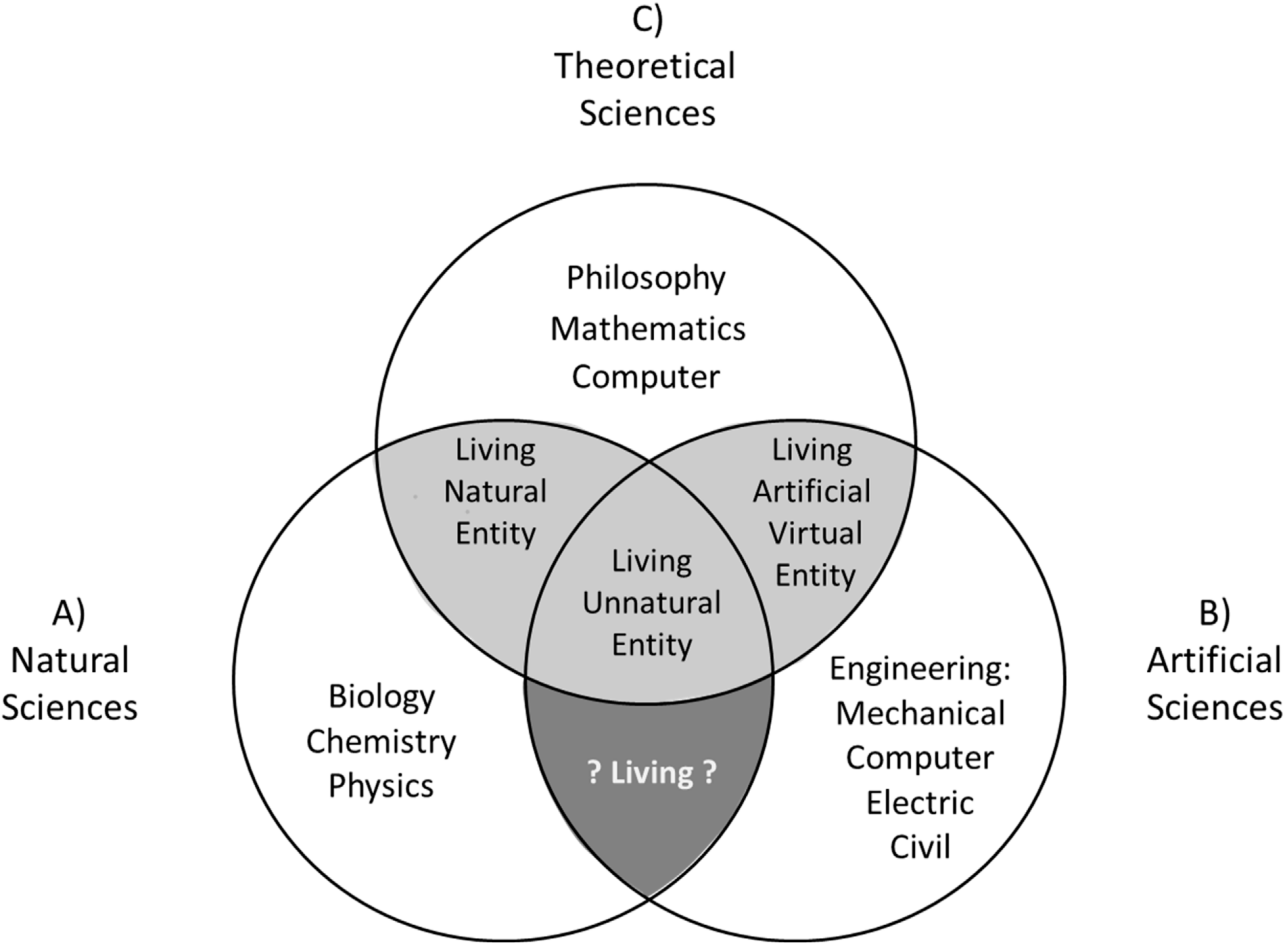
Interdisciplinarity of synthetic biology. Three main spheres to study living entities: **(A)** Natural Sciences (e.g.,: biology, genetics, microbiology, chemistry, physics … ); **(B)** Artificial Sciences (e.g.,: mechanical, computers, electric and civil engineering); **(C)** Theoretical sciences (e.g.,: philosophy, mathematics, computer … ). Light gray areas are the intersection of spheres of study where we could build knowledge on the living entities. Dark gray area is the intersection of spheres of study where we could not build knowledge on living entities.

Inspired by Biglan’s classification, I propose an interdisciplinary model for synthetic biology that articulates three general spheres of science, which include all the specific disciplines involved in the project of studying the object of living entities in a collaborative way (Figure 3):1- « Natural science » (which includes biology, microbiology, genetic, chemistry; Figure 3A), corresponding to the “hard” disciplines of Biglan; 2- « Artificial science » (which includes all types of engineering: civil, electric, mechanic, informatics; Figure 3B), corresponding to the “apply” disciplines of Biglan; 3- « Theoretical sciences » (which includes mathematics and computer sciences; Figure 3C), corresponding to “pure” disciplines of Biglan. This interdisciplinary dynamic in synthetic biology is well represented by the design of the “genetic toggle switch” module (Gardner et al., 2000, p.339): « *Here we have integrated theory and experiment by constructing and testing a synthetic, bistable gene* [natural science] *circuit* [artificial science] *based on the predictions of a simple mathematical model* [theoretical science] ».

Compared to Biglan first and second dimensions, I choose to separate “natural science” and “artificial science” in my model considering the structural origins (or composition) of the object studied in synthetic biology (as presented previously in this manuscript: Figure 2, Level 2). I also prefer the term “theoretical science” over “humanities” in order to blend mathematics and computer sciences with humanities disciplines in a pragmatic theoretical context. I would also include the discipline philosophy (philosophy of science and philosophy of biology) in the sphere of “theoretical sciences”, considering, on the one hand, that it could help clarify the objects and objectives of a given discipline, as I am doing with the present manuscript, on the other hand, it could contribute to solving concrete scientific problems (as exposed by Laplane et al., 2019)^10^. Indeed, one of the main concrete problems for synthetic biology is linked to its capacity to build new entities characterized as “living”, even though the concept of “living entity” has no clear definition and is still debated within the communities of philosophers and scientists (as I will expose in the last section of this manuscript; Trifonov, 2011). How can we build and characterize an object as “living” if we do not have a clear definition of it?

Considering the fundamental role of philosophy (theoretical sciences) in establishing a definition of the concept “living” and identifying living entities, we could add at the intersections of the three spheres of the model whether the constructed entity is characterized as “living” or “non-living”. In doing so, the light gray areas in my model correspond to the intersections of the spheres where we could identify and construct knowledge about living entities according to philosophy (between Theoretical Sciences and Natural Science and between Theoretical Sciences and Artificial Science). The dark gray area in my model corresponds to the intersection of the spheres where we could not construct knowledge about living entities (between Natural Science and Artificial Science) since philosophy (included in Theoretical Sciences) is not involved in this intersection.

This aspect of my model is different from the third dimension proposed by Biglan’s model, since the latter does not specify the definition (or meaning) of the “living” concept he used to classify his disciplines. For example, a chemical system classifies as a “non-living system” by Biglan could be characterized as living using different or flexible definitions of the concept “living”, like the definition use by NASA (Joyce, 1995): « *Self-sustaining chemical system capable of Darwinian evolution* ». More fundamentally, Biglan classify the discipline philosophy in the “non-living system” group, despite the fact that philosophy is at the center of the epistemology of the concept “living”, and that it might play a main role to identify which entities are living and non-living, as expose by my model with the gray intersections (Figure 3).

Thus, “Theoretical Sciences”, which have been dissociated from biology for several decades, would regain their place alongside the experimental sciences through the development of synthetic biology (as also affirm by Morange, 2009, p22-23). More generally, this model contributes to the dialogue between the “two academic cultures” (to use the expression use by Snow, 2001), which are the natural sciences and the humanities.

Following and related to this first fundamental objective of synthetic biology on interdisciplinary collaboration, essential to the resolution of complex problems, I present in the next sections two other fundamental objectives of this discipline, representing complex problems where synthetic biology could contribute: Developing knowledge on living entities, Developing a pragmatic definition of the concept of “living".

### 5.2 Knowledge on living entities

Each biological subdiscipline has its own angle or structural hierarchical level of study of living entities, shedding light on one facet of the living world. By pooling this knowledge, we can have a better understand of the living. For example, molecular biology and genetics study the DNA molecule, biochemistry study the metabolism of living organisms, microbiology and cellular biology study the cell, ecology study populations, communities and ecosystems. Synthetic biology has for its part the particular capacity of constructing living entities (Forster and Church, 2007; Ruiz-Mirazo and Moreno, 2013), where “constructing” can be synonymous of “knowing”, as suggested by Keller (2009, p.337): “*Making, be it with mathematical objects, paper tools, chemical precipitates, or nucleo-protein or robotic modules-as itself a form of knowing*”. Considering the range of living entities that can be constructed by synthetic biology (Figure 2, Level 1), the second fundamental objective of synthetic biology should then be the development of knowledge on living entities, in collaboration with other biological subdiscipline. We could point out four kinds of knowledge that could be developed by synthetic biology.

First, synthetic biology has the potential to contribute to the development of knowledge on living entities found in the present or past Nature, as outlined previously with the construction of living entities made from natural structures (Figure 2, Level 2: A) that support natural functions (Figure 2, Level 3: I). We could include in this category of knowledge the research on the origin of Life on Earth (Oparin, 1924; Fox and Dose, 1972; Miller and Orgel, 1974; Dunér et al., 2016) and on the “Last universal common ancestor (LUCA) " (Rouch, 2014; Weiss, 2016; Weiss et al., 2018).

Second, this discipline has the capacity to imagine and construct living entities that could have appeared in Nature following alternative path of evolution (Keller, 2009, p.337): « [*…*] *manual and experimental manipulations can stir and provoke the theoretical imagination, thus leading us to an understanding not of how life actually did evolve, but how it could have evolved* ». This aspect reminds us of the “videotape” metaphor of Gould (1989), who argues that if we could rewind the movie of the history of life and replay it, the evolutionary history that would unfold would be different, with different living entities in a different tree of life. We could therefore imagine and build these possible alternative living entities in the laboratory, made from natural and/or non-natural organic structures (Figure 2, Level 2: A and B), which can support natural, supernatural and/or non-natural functions (Figure 2, Level 3: I, II and III).

Third, we could use synthetic biology to imagine and build living entities that would come from outside the Earth’s biosphere, from elsewhere in the Universe. Let us underline that NASA founded a major Astrobiology Institute^11^ in 1998, whose goal is to answer these three fundamental questions: “*How did life begin and evolve? Is there life elsewhere in the Universe? What is the future of life on Earth and in the* Universe? That said, one of the great challenges of the discipline astrobiology lies in the fact that it is still looking for its own object of study, extraterrestrial living entities, which may have structures and functions different from those observed in terrestrial Nature (Dick, 2012; Cleland, 2019; Kolb, 2019; Malaterre et al., 2022; Malaterre and Lareau, 2023). Synthetic biology can contribute in studying the structural and functional flexibility and possibility of the living entities in the laboratory, as points out by Benner (2013, p.118): “*Synthetic biology should also help NASA to seek life in its probes of the Solar System. By asking what is possible in the chemistry that supports life, we are more likely to recognize weird life should we encounter it*".

That said, a fundamental question remains: what is the definition of the concept “living entity”, or to put it more simply, “what is life"^12^ ? Indeed, when building new living entities (in synthetic biology) or searching for new living entities in the Universe (in astrobiology), we first need a pragmatic definition of this concept. This question is another fundamental objective that synthetic biology could contribute to, linked to the previous objectives of interdisciplinary collaboration and development of knowledge, as I expose in the next section.

### 5.3 A pragmatic definition of the concept of “living"

Scientists and philosophers still do not agree on a clear and unanimous definition of “living”: There are 123 non-redundant definitions of “living” (Trifonov, 2011). Each discipline has its own angle and agenda depending on their theoretical and experimental perspective, leading to forge definitions of living in a subjective, redundant, or divergent manner depending on their disciplinary agenda (Machery, 2012; Bich and Green, 2018). This definitional pluralism can become a problem in certain situations requiring a more consensual and practical definition of “living entity”, as exposed for synthetic biology and exobiology.

In order to overcome this complex epistemological problem, it is necessary to use a collaborative and interdisciplinary approach (Dupré and O’Malley, 2009; Ruiz-Mirazo et al., 2010; Cleland, 2012). Considering its internal and external interdisciplinarity (corresponding to the first objective; Figure 3), its wide range of building entities (corresponding to the second objective; Figure 2) and its goal of building the minimal cellular living entity following different methods (Figure 1), synthetic biology could play a central role in defining the concept of living, and thus should represent its third fundamental objective (Morange, 2009, p.29): « *Synthetic biologists will answer the question ‘What is life?’ and give an implicit definition of it* ». I present in this section three successive steps to achieve this objective.

First, we must ensure that we have a representative heterogenous sample of living entities, in plurality and variety, to extract a definition of the concept of “living entity”. In doing so, the study of the terrestrial tree of life (including present and past species) is not sufficient in this epistemological quest, considering that it represents only one example of life (n = 1), as Cleland (2012, p.126) states: “*One cannot safely generalize to all of life, wherever and whenever it may be found, from a single, potentially unrepresentative, example of life*”. Indeed, all terrestrial living entities derive from the present evolutionary tree and from the same ancestor or group of ancestors (Doolittle, 1999; Bapteste et al., 2009). We must therefore imagine alternative trees of life, search for extraterrestrial living entities, and test the flexibility of life to increase the number of examples of life, as exposed previously with the second objective of synthetic biology. Besides natural entities, we could also conceive living entities made of inorganic or immaterial structures (such as electrical and computer circuits, mechanical systems or computer software) that would artificially and virtually mimic the minimal living functions, as expose previously with the objects of synthetic biology (Figure 2). These types of entities could in turn contribute to studying and defining the concept of “living entity".

**FIGURE 4:**
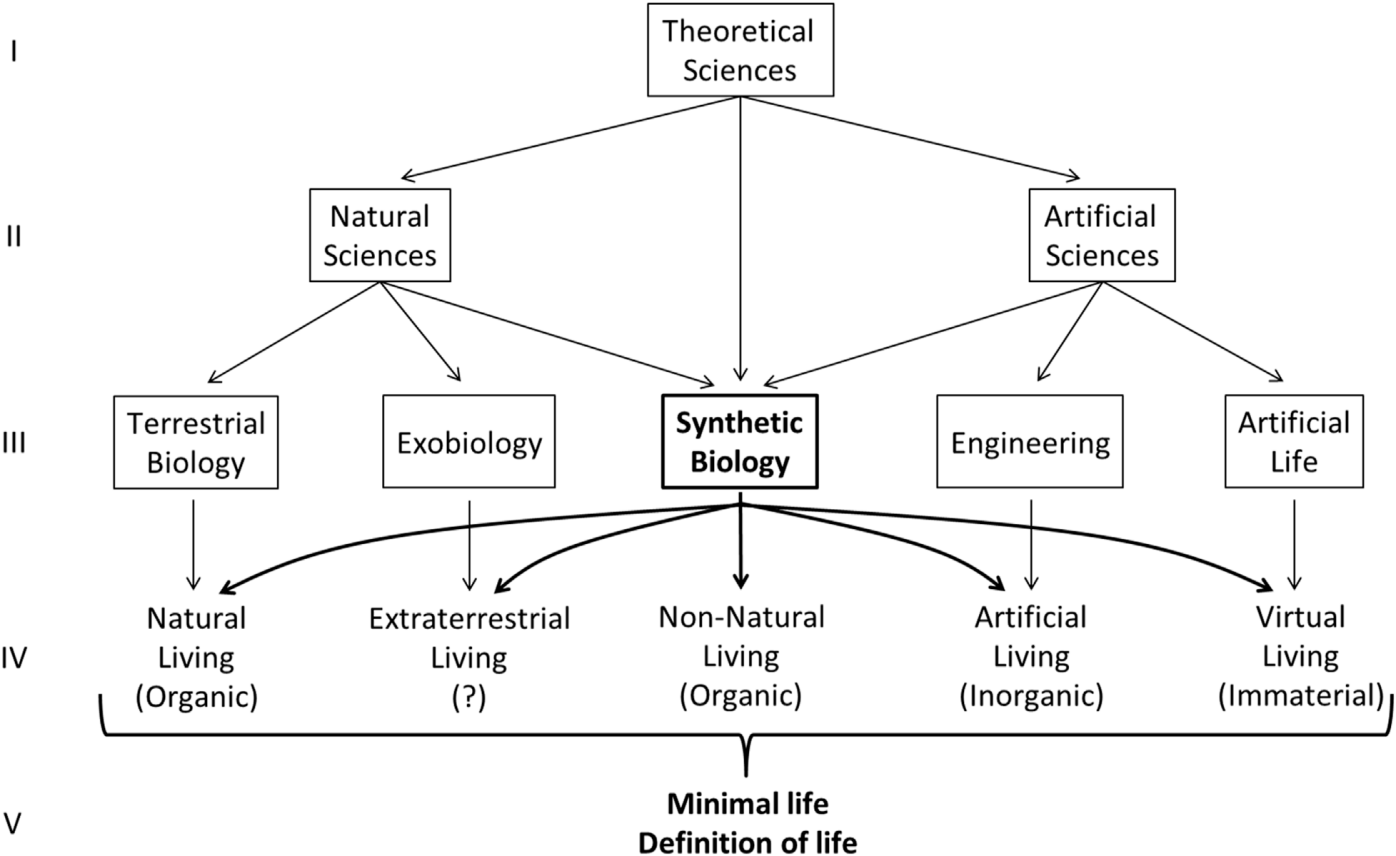
Collaboration on the epistemology of the living. Raw I and II: Theoretical sciences are necessary to help Natural and Artificial Sciences identify their objectives of the object of study: living entities. Raw II and III: Examples of specific disciplines in Natural Sciences (Terrestrial biology and exobiology) and Artificial Sciences (Engineering and Artificial life). Synthetic biology is half-natural and half-artificial science considering its interdisciplinary foundation (on methods and objects of study). Raw III and IV: each specific disciplinary can study living entities through their methods and point of view. Synthetic biology, considering its interdisciplinary foundation, can collaborate on both sides of these sciences (Natural and Artificial) and make bridges between them. On raw IV and V: All specific disciplines can collaborate to establish the minimal living entity and the definition of life.

This objective requires, as exposed previously, the collaboration of all the disciplines (Figure 3), from Natural, Artificial and Theoretical sciences, which study different aspects of the living at the structural and functional level (Figure 4, I, II, III, IV). Synthetic biology, being between natural and artificial sciences, can be the interdisciplinary bridges of these disciplines for the study of the diversity and flexibility of living entities, considering its ability to build (and develop knowledge on) different kinds of living entities.

Second, from this plurality and diversity of living entities, we could then extract or distill a minimal entity common to all these living entities (Figure 4, IV, V). This minimal entity has potentially never existed naturally by itself in its most refined form but would be found in an immutable way at the heart of all entities characterized as living. It would be buried under different natural structural (Figure 2, Level 2, A) and functional (Figure 2, Level 3, I) layers, resulting from the natural evolution process in different environmental contexts (Glass et al., 2006, p.425): “*In an environment that is free from stress and provides all necessary nutrients, what would constitute the simplest free-living organism*?

Third, once the minimal living entity is “extracted”, we could verify if we can make this natural minimal living even more minimalism by replacing a natural module supporting a given function by another laboratory-designed module (of natural or non-natural structural origins; Figure 2, level 2) that could be simpler and/or more efficient than the naturally evolved module (like supernatural functions; Figure 2, level 3, II). For example, the studies on rewriting a genome by altering its genetic code (Ostrov et al., 2016; Fredens et al., 2019).

Considering these three steps, the minimal living entity generated could be considered as the materialization or a pragmatic definition of the concept “living entity".

## 6 Conclusion

I present in this manuscript an epistemological analysis of synthetic biology to specify its objects and objectives, in order to define and develop this new discipline.

I first present and investigate the pluralism of “research programs” developed at the foundation of synthetic biology, complemented by the analysis of a scientometric study, in order to establish an overview of the practices and concepts at the foundation of this new emerging discipline.

Then, to better define synthetic biology through its object of study, I suggest a three-level classification of living entities that can be built in the laboratory, based on their hierarchical structural level (molecule, cell, population-community, ecosystem-biome, biosphere), structural origin (natural, non-natural, artificial, virtual) and functional origin (natural, supernatural, non-natural).

Finally, considering its objects of study, I propose three successively linked objectives in which synthetic biology can contribute: to maintain internal and external interdisciplinary collaboration (between natural, artificial, and theoretical sciences); to develop knowledge on living entities (by building a great variety of them); to establish a pragmatic definition of the concept of “living” (by distilling the minimal living organism).

These fundamental objects and objectives could correspond to a new theoretical framework or “research program” for synthetic biology, which defines this new discipline. Thus, considering this epistemology, we might conclude that synthetic biology is a discipline at his “coming of age” step (rather than at his “child” step, as affirm by Bensaude Vincent (2013), p.128), which has the potential to develop a new and distinct angle for studying, understanding, and defining living entities in a collaborative manner.

## Data Availability

The original contributions presented in the study are included in the article/Supplementary material, further inquiries can be directed to the corresponding author.

## Footnotes

1 The first scientist to use the term “synthetic biology” was probably Stéphane Leduc in his book *Synthetic Biology* published in 1912, which exposed the creation of non-living entities that could reproduce certain structures and functions of living entities (Keller, 2009, p.335). Today, it is generally recognized that the term made its appearance with the meaning used today following the publication of an important article by Hartwell in 1999 (titled “*From molecular to modular cell biology”* and publish in the journal *Nature*), which laid the foundations of this potential new discipline (Morange, 2009, p.22).

2 “Paradigm” and “Research program” refer respectively to the theory of knowledge developed by Thomas Kuhn (1962) and Imre Lakatos (1968-1969). A Kuhnian paradigm is, as summarized by Godfrey-Smith (2003), p.239): " *a whole “way of doing science”. A paradigm will typically include theoretical ideas about the world, methods, and subtle habits of mind and standards used to assess “good work” in the field*”. A Lakatosian research program is, for its part (Godfrey-Smith, 2003, p.240): " *a sequence of scientific theories that all explore and develop the same basic theoretical ideas* ". I favor the Lakatosian concept of “Research program” *(complemented by the “tradition of research” of Laudan (1977))* which allows the coexistence of several progressive “programs” at the same time, as I will expose in this section for synthetic biology, while the khunian paradigm approach only allows one paradigm at the time, which can be replaced completely through a “revolution” process.

3 This study follows another scientometric study publishes by Oldham et al. (2012), which analyze 1,255 articles publish between 1980 and 2011.

4 46.5% of the articles on synthetic biology, as well as 8 of the 10 most important institutions publishing in the field of synthetic biology, are of American origin. American scientists pioneers of synthetic biology include C.A. Voigt, D. Endy, J.M. Church, J.D. Keasling, J.J. Collins and J.C. Venter.

5 Some scientists are trying to broaden the field of synthetic biology by including engineer (or design) proteins (Foo et al., 2012; Liu, 2015; Hu, 2015; Chen and Elowitz, 2021), but this object is still at the hierarchical level of molecules, and closely connected to DNA considering their link to the transcription-traduction process.

6 Some entities from different levels of structural hierarchy of the living are considered by some scientists and philosophers as “superorganisms” following revision of certain properties. For examples: biofilms (Shapiro, 1988; Watnick and Kolter, 2000; O'Malley and Dupré, 2007), clonal plant populations (such as trembling aspen; Jackson et al., 1986; Bouchard, 2008), eusocial animal population (such as the termites; Wilson, 1985; Turner J. S., 2000; Bouchard, 2010), the whole biosphere (Lovelock, 1979; Doolittle, 2017). These “superorganisms” could be objects of study for synthetic biology.

7
https://www.bostondynamics.com/atlas

8 There is a debate in philosophy of biology on reductionism in biology. I will not elaborate on this topic in the present article. To read on this subject, I recommend the following articles: Hull (1972) Reductionism in Genetics—Biology or Philosophy? Gilbert and Sarkar (2000) Embracing complexity: organicism for the 21st century; Rosenberg (2006) *Darwinian Reductionism: Or, How to Stop Worrying and Love Molecular Biology*; Morange (2006) Post-genomics, between reduction and emergence; Brigandt (2010) Beyond reduction and pluralism: toward an epistemology of explanatory integration in biology; Muszynski and Malaterre (2021) A roadmap to explanatory pluralism: Introduction to the topical collection.

9 Biglan was inspired by the theory of knowledge of Thomas Kuhn and his concept of “paradigm” (a body of theory which is subscribed to by all members of the field). He then characterizing is “hard” and “soft” disciplines respectively as “paradigmatic” (with high degree of paradigmatic consensus) and “pre-paradigmatic” (paradigms that are more nebulous). As I previously mentioned in this paper (footnote 2), I prefer the term “research programs” of Imre (Lakatos, 1968-1969) over the “paradigm” of Thomas Kuhn, considering that it is more adapted to an interdisciplinary and pluralistic context of research projects.

10 We could also include the discipline of ethics in this sphere since all science discipline will need ethical guidance and boundaries it their research. On that matter, synthetic biology is currently the subject of several discussions (which I will not elaborate in the present paper) on the limits of what we can build or modify in living entities in order to preserve the integrity of life and ensure our biosafety (Cello et al., 2002; Samuel et al., 2009; PCSBI, 2011; Keiper and Atanassova, 2020; Lewis et al., 2020).

11 Astrobiology Institut of NASA: https://astrobiology.nasa.gov/nai/about/

12 The book “What is life ? the physical aspect of living cell”, published in 1944 by Erwin Schrödinger (1944), laid the modern foundations for this fundamental and still relevant questioning.
